# Increase in power by obtaining 10 or more controls per case when type-1 error is small in large-scale association studies

**DOI:** 10.1186/s12874-023-01973-x

**Published:** 2023-06-29

**Authors:** Hormuzd A. Katki, Sonja I. Berndt, Mitchell J. Machiela, Douglas R. Stewart, Montserrat Garcia-Closas, Jung Kim, Jianxin Shi, Kai Yu, Nathaniel Rothman

**Affiliations:** Division of Cancer Epidemiology and Genetics, Department of Health and Human Services, National Cancer Institute, National Institutes of Health, Bethesda, MD USA

**Keywords:** Control selection, Multiple comparisons, Study design, Molecular epidemiology

## Abstract

**Background:**

The rule of thumb that there is little gain in statistical power by obtaining more than 4 controls per case, is based on type-1 error α = 0.05. However, association studies that evaluate thousands or millions of associations use smaller α and may have access to plentiful controls. We investigate power gains, and reductions in *p*-values, when increasing well beyond 4 controls per case, for small α.

**Methods:**

We calculate the power, the median expected *p*-value, and the minimum detectable odds-ratio (OR), as a function of the number of controls/case, as α decreases.

**Results:**

As α decreases, at each ratio of controls per case, the increase in power is larger than for α = 0.05. For α between 10^–6^ and 10^–9^ (typical for thousands or millions of associations), increasing from 4 controls per case to 10–50 controls per case increases power. For example, a study with power = 0.2 (α = 5 × 10^–8^) with 1 control/case has power = 0.65 with 4 controls/case, but with 10 controls/case has power = 0.78, and with 50 controls/case has power = 0.84. For situations where obtaining more than 4 controls per case provides small increases in power beyond 0.9 (at small α), the expected *p*-value can decrease by orders-of-magnitude below α. Increasing from 1 to 4 controls/case reduces the minimum detectable OR toward the null by 20.9%, and from 4 to 50 controls/case reduces by an additional 9.7%, a result which applies regardless of α and hence also applies to “regular” α = 0.05 epidemiology.

**Conclusions:**

At small α, versus 4 controls/case, recruiting 10 or more controls/cases can increase power, reduce the expected *p*-value by 1–2 orders of magnitude, and meaningfully reduce the minimum detectable OR. These benefits of increasing the controls/case ratio increase as the number of cases increases, although the amount of benefit depends on exposure frequencies and true OR. Provided that controls are comparable to cases, our findings suggest greater sharing of comparable controls in large-scale association studies.

**Supplementary Information:**

The online version contains supplementary material available at 10.1186/s12874-023-01973-x.

## Introduction

A well-known rule of thumb in epidemiology is that there is little gain in statistical power by obtaining more than 4 controls per case [1–7]. Well-known exceptions to this rule are situations with rare exposures or large odds-ratios [1–7]. However, it may be less well-known that the rule presumes type-1 error α = 0.05. Large-scale association studies examine thousands or millions of markers, but use small type-1 error α and small *p*-value thresholds to declare statistical significance [8, 9]. For example, exome-wide or genome-wide association studies (GWAS) examine millions of variants but require small α, such as α = 2.5 × 10^–6^ for a gene-based burden test [10] and α = 5 × 10^–8^ or 5 × 10^–9^ for a single-marker-based test [11, 12]. Sometimes, plentiful appropriate controls are potentially available (such as consortial, registry, or cohort studies) or may be borrowed at little, or no, cost from other studies. When the number of cases is fixed, obtaining as many “free” controls as possible is ideal [1–7]. However, controls are rarely literally “free”. It is well-known that the cost-effective number of controls depends on the ratio of costs of obtaining controls vs cases [1–7], although it is often hard to quantify “costs”.

A salient example of power gains by increasing beyond 4 controls/case is GWAS [13–16]. Methods have been proposed to borrow appropriate controls across GWAS [17–22] or genotype all cohort/biobank members [23–25], either of which has resulted in a large number of controls/case for GWAS of many diseases [26–32]. Although important, GWAS research has focused on a single α (“genome-wide significance”), not how statistical power varies with the controls/case ratio as α varies. The general epidemiologic principles justifying increasing beyond 4 controls/case have not been articulated.

For planning a single large-scale association study, we examine how power increases, and the median expected *p*-value decreases, with increasing number of appropriate controls/case as a function of α. Particularly, we examine the value of obtaining > 4 controls/case for 2 situations: (1) when 4 controls/case is under-powered, to identify novel associations, or (2) when 4 controls/case is well-powered, to provide greater evidence for association by further reducing the *p*-value. We also derive the decrease in minimum detectable odds-ratio as the number of controls/case increases. We apply our findings to choosing the number of controls/case for a GWAS.

## Methods

We focus on testing for association via an odds-ratio in a case–control design. Our general findings are based on the classical calculation of the increase in statistical efficiency with increasing controls/case [1–7]. This presumes “local alternatives”: as the number of cases increases, the minimum odds-ratio to detect becomes closer to 1 [1–7]. This asymptotic is apt when studies with larger sample sizes seek to identify smaller effects. The value of the local alternatives asymptotic is to not require individually specifying numbers of cases or controls, marker prevalence, or odds-ratio (OR) – these variables are subsumed by solely specifying the power for a study with 1 control/case. Although the equations demonstrate general principles, for a specific application it is of interest to examine the interplay of all variables, which we will do for GWAS. Here we show the final equations – see Supplement for derivations.

### Increase in power with increasing number of controls/case by α

Denote power for an association with 1 control/case as 1-β_1_ and power with J controls/case as 1-β_J_. The power for J controls/case is (derivation in Supplement)

1 $$1-{\beta }_{J}=\Phi \left({Z}_{\left\{1-{\beta }_{1}\right\}}+\left\{{{Z}_{\left\{1-{\beta }_{1}\right\}}+Z}_{\left\{1-\frac{\alpha }{2}\right\}}\right\}\left\{\sqrt{\frac{2J}{J+1}}-1\right\}\right),$$

where $\Phi$ is the CDF of the standard normal distribution and ${Z}_{\left\{x\right\}}$ is the upper two-sided standard normal deviate at level $x$; for example ${Z}_{\left\{1-(5e-08)/2\right\}}=5.45$ and ${Z}_{\left\{0.8\right\}}=0.84$. The key point is that the power increases as ${Z}_{\left\{1-\alpha /2\right\}}$ increases, i.e., as α decreases. Thus, fixing the power of a study with 1 control/case (regardless of the combination of sample-sizes, marker prevalences, and odds-ratio that results in that power), the increase in power for J controls/cases, versus 1 control/case, is greater as α decreases. We will plot this power for J controls/case as J increases, for various α, to demonstrate that the increase in power from J controls/case increases as α decreases.

However, note that Eq. (1) will not tell us about power for a particular study where we must set exposure frequencies, sample sizes, and other parameters. Figures 3, S1, and S2 vary all parameters of a traditional power calculation to examine power for particular GWAS studies (see Supplement for derivation):

$${Z}_{\left\{1-\beta \right\}}=\frac{\sqrt{2N}|{p}_{1}-{p}_{2}|-{{\phi }_{0}Z}_{\left\{1-\alpha /2\right\}}}{{\phi }_{1}},$$

where $N$ is the total sample size (total number of alleles), ${p}_{1}$ and ${p}_{2}$ are exposure frequencies for cases and controls (respectively), and ${\phi }_{0}$ and ${\phi }_{1}$ are the standard deviations under the null and alternative hypotheses (respectively). Parenthetically, the Genetic Association Study (GAS) Power Calculator [13, 33] substitutes the variance under the alternative for the variance under the null (i.e. substitutes ${\phi }_{1}$ for ${\phi }_{0}$), which is appropriate for a Wald test, but not for a score test (see Supplement).

### Reduction in median expected *p*-value with increasing number of controls/case by α

*P*-values are random variables that vary over hypothetical study replications [34, 35]. The *p*-value estimates the median of the *p*-values in a hypothetical population of all possible study results [36]. That is, under the null hypothesis of no effect, *p*-values have a uniform distribution, but under the alternative hypothesis, the distribution of *p*-values is highly skewed toward 0 and the median expected (two-sided) *p*-value [36] is

2 $$p=2\times\Phi (-{|Z}_{\left\{1-\beta \right\}}+{Z}_{\left\{1-\alpha /2\right\}}|).$$

**Fig. 1:**
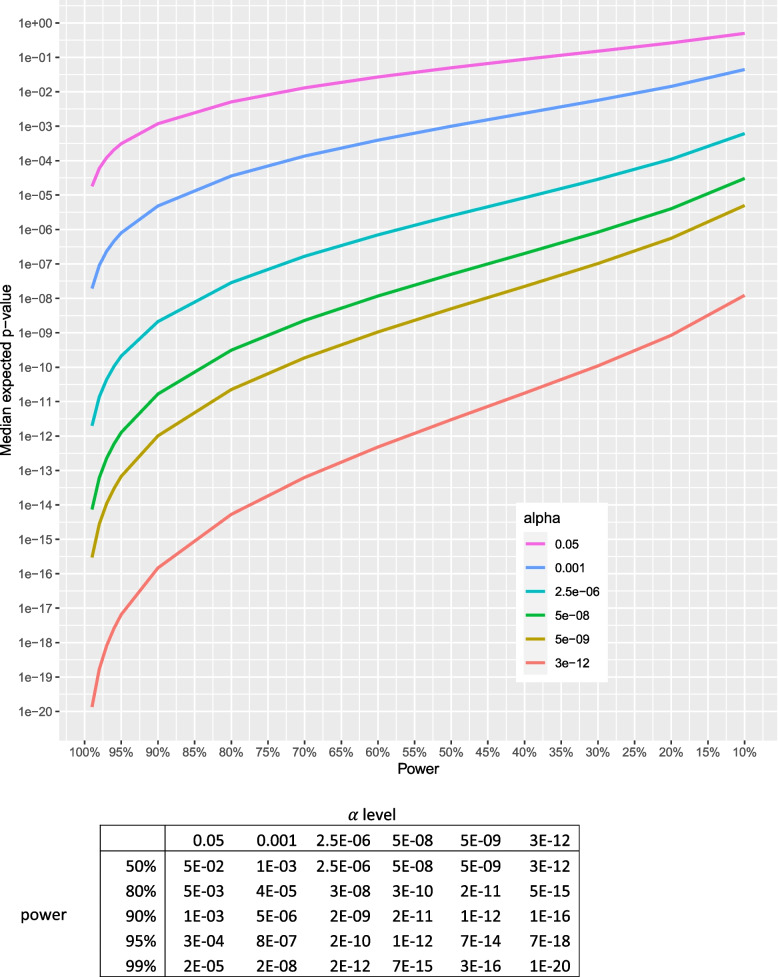
Median expected *p*-value versus power at selected α. Table below figure shows the median expected *p*-value for chosen powers and α. Note that when power = 50%, the median expected *p*-value equals α

The median expected *p*-value informs about the *p*-value that can be expected for an association with given power and α. Figure 1 plots the median expected *p*-value versus power at selected α. For an association with 50% power, the median expected *p*-value equals α. At 80% power and α = 0.05, the median expected *p*-value for the association is 0.0051. At 80% power and genome-wide significant α = 5 × 10^–8^, the median expected *p*-value is 3 × 10^–10^. See Supplement for more explanation.

The median expected *p*-value for a study with J controls/case is calculated by plugging in the power from Eq. (1). The key point is that the median expected *p*-value decreases as α decreases. Thus, fixing the power of a study with 1 control/case (regardless of the combination of sample-sizes, marker prevalences, and odds-ratio that results in that power), the median expected *p*-value for J controls/cases, versus 1 control/case, is lower as α decreases. We will plot the median expected *p*-value for J controls/case as J increases, for various α, to demonstrate that the reduction in power from J controls/case increases as α decreases.

### Fractional reduction, toward the null, in the minimum detectable OR, regardless of α

Denote the minimum detectable OR for a study with 1 control/case as OR_1_, and for a study with J controls/case as OR_J_. The maximum reduction in the OR toward the null is OR_1_-1, of which a study with J controls/case will achieve OR_1_-OR_J_. The fractional reduction, toward the null, in the minimum detectable OR by obtaining J controls/case is approximately (derivation in Supplement)

3 $$\frac{{OR}_{1}-{OR}_{J}}{{OR}_{1}-1}\approx 1-\sqrt{\frac{J+1}{2J}}.$$

Note that the fractional reduction, toward the null, in the minimum detectable OR depends only on the number of controls/case. Power, α, marker frequencies, and sample sizes are all subsumed into the minimum detectable OR for 1 control/case. Because even α is subsumed, this result applies also to “regular” α = 0.05 epidemiology. Hence, once one has calculated the minimum detectable OR for 1 control/case, the equation above calculates the minimum detectable OR for any number of controls/case.

In particular, as J controls/case increases, the fractional reduction, toward the null, in the minimum detectable OR asymptotes at $1-\sqrt{1/2}=29.3\%$. For example, if the minimum detectable OR for a study with 1 control/case were 2, then the minimum detectable OR for a study with large number of controls/case is $2-0.293\left(2-1\right)=1.71$. We will make a table showing how the minimum detectable OR is reduced as the number of controls/case increases.

## Results

### Increase in power with increasing number of controls/case by α

**Fig. 2:**
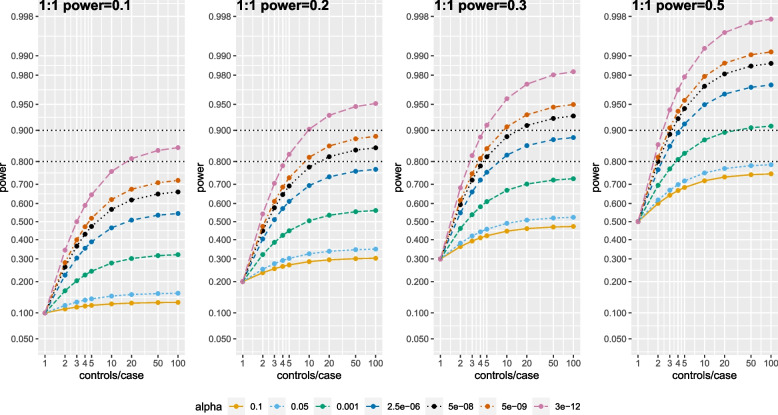
Power of an association versus the number of controls per case as α decreases, as the power with 1 control per case increases from 0.1 to 0.2 to 0.3 to 0.5. Y-axis is on the probit scale to appropriately show meaningful differences in seemingly small power gains beyond 0.9; x-axis is on a log scale. Dotted horizontal lines indicate powers of 0.8 and 0.9

Figure 2 and Table S1 show power for an association with J controls/case as α decreases (based on Eq. (1)), fixing power for the association with 1 control/case to be low/moderate (0.1–0.5). For α = 0.05, the power does not increase much as controls increase beyond 4 in any situation, agreeing with classical results [1–7]. But this is not true for α = 0.001, and especially gene-based-significance (α = 2.5 × 10^–6^), genome-wide-significance (α = 5 × 10^–8^), or for an example “stringent” threshold (α = 3 × 10^–12^). The increase in power from each value of controls/case is stronger as α decreases and when a study with 1 control/case has low power for the association.

For example, when a 1 control/case study has power = 0.2 and α = 0.05, the power asymptotes at 0.35 for 100 controls/case. For smaller α, obtaining 4 controls/case raises power from 0.2 to 0.57 (α = 2.5 × 10^–6^), 0.65 (α = 5 × 10^–8^), 0.69 (α = 5 × 10^–9^), and 0.78 (α = 3 × 10^–12^). However, power remains < 0.8. Further increasing from 4 to 50 controls/case raises power from 0.57 to 0.76 (α = 2.5 × 10^–6^), 0.65 to 0.84 (α = 5 × 10^–8^), 0.69 to 0.88 (α = 5 × 10^–9^), and from 0.78 to 0.95 (α = 3 × 10^–12^). As expected, 4 controls/case raises power, but increasing to 50 controls/case provides additional gains to approach or exceed power = 0.8 or 0.9. Similar gains in power are observed for obtaining 50 vs. 4 controls/case when 1 control/case has power 0.1–0.3.

### Reduction in median expected *p*-value with increasing number of controls/case by α

**Fig. 3:**
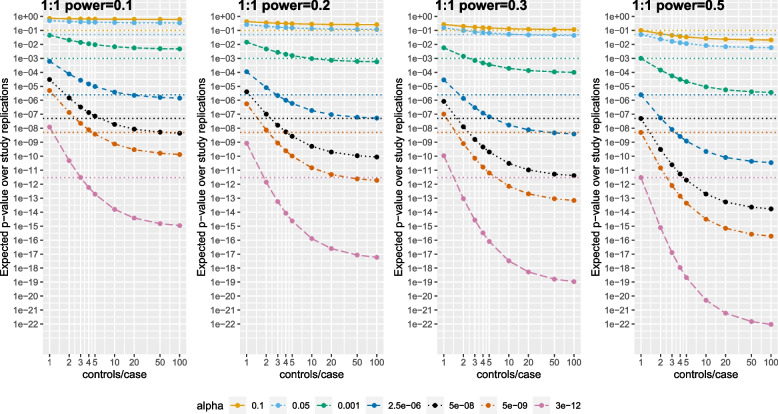
Expected *p*-value under replication of an association versus the number of controls per case as α decreases, as the power of a study with 1 control per case increases from 0.1 to 0.2 to 0.3 to 0.5. Dotted lines represent the α level for that color. Both axes are on a log scale

Figure 3 and Table S2 show the median expected *p*-value for an association (based on Eq. (2)) decreasing as the number of controls/case increases, given α and power for the association in a 1 control/case study. For α = 0.05, increasing controls/case does not substantially decrease the median expected *p*-value. In contrast, as α decreases, increasing controls/case beyond 4 decreases the median expected *p*-value by orders of magnitude, with the ratio reduction increasing as α decreases.

For example, when a 1 control/case has power = 0.1, 1 vs. 4 controls/case reduces the median expected *p*-value 60-fold (*p* = 6 × 10^–4^ to *p* = 1 × 10^–5^; α = 2.5 × 10^–6^), by 300-fold (*p* = 3 × 10^–5^ to *p* = 1 × 10^–7^, α = 5 × 10^–8^), by 600-fold (*p* = 5 × 10^–6^ to *p* = 8 × 10^–9^; α = 5 × 10^–9^), and by 16,000-fold (*p* = 1 × 10^–8^ to *p* = 6 × 10^–13^; α = 3 × 10^–12^). However, the median expected *p*-value remains above α. Further increasing from 4 to 50 controls/case, the median expected *p*-value drops to below α, reducing by 5-fold (*p* = 1 × 10^–5^ to *p* = 2 × 10^–6^; α = 2.5 × 10^–6^), by 20-fold (*p* = 1 × 10^–7^ to *p* = 5 × 10^–9^, α = 5 × 10^–8^), by 40-fold (*p* = 8 × 10^–9^ to *p* = 2 × 10^–10^; α = 5 × 10^–9^), and by 300-fold (*p* = 6 × 10^–13^ to *p* = 2 × 10^–15^; α = 3 × 10^–12^). Thus, obtaining 4 controls/case reduces median expected *p*-values, but sometimes not below α. Increasing to 50 controls/case further reduces median expected *p*-values by 5-fold to 300-fold, and in this situation, to below α, potentially identifying novel associations.

When power = 0.5 for a 1 control/case study, power is > 0.89 with J = 4 controls for α α ≤ 2.5 × 10^–6^ (Fig. 2), which represent well-powered associations. Thus increasing from 4 to 50 controls/case cannot increase power by much, yet, the median expected *p*-value still decreases by orders of magnitude (Fig. 3). This is because small increases in power > 0.9 substantially reduce the expected *p*-value (Fig. 1), providing concomitantly greater reassurance that an association is not a false-positive.

For example, when 1 control/case has power = 0.5, increasing from 1 to 4 controls/case reduces the median expected *p*-value 1,000-fold (*p* = 2 × 10^–6^ to *p* = 3 × 10^–9^; α = 2.5 × 10^–6^), by 10,000-fold (*p* = 5 × 10^–8^ to *p* = 5 × 10^–12^, α = 5 × 10^–8^), by 50,000-fold (*p* = 5 × 10^–9^ to *p* = 1 × 10^–13^; α = 5 × 10^–9^), and by 3,000,000-fold (*p* = 3 × 10^–12^ to 1 × 10^–18^; α = 3 × 10^–12^). These already represent substantial *p*-value reductions, sometimes below a “stringent” *p* = 3 × 10^–12^ threshold.

Moreover, substantial further reductions are possible by increasing from 4 to 50 controls/case. For α = 2.5 × 10^–6^, power increases only from 0.894 to 0.970, but the median expected *p*-value decreases 75-fold (*p* = 3 × 10^–9^ to *p* = 4 × 10^–11^). For α = 5 × 10^–8^, power increases only from 0.926 to 0.985, but the median expected *p*-value decreases 235-fold (*p* = 5 × 10^–12^ to *p* = 2 × 10^–14^). For α = 5 × 10^–9^, power increases only from 0.939 to 0.990, but the median expected *p*-value decreases 300-fold (*p* = 1 × 10^–13^ to *p* = 3 × 10^–16^). For α = 3 × 10^–12^, power increases only from 0.968 to 0.997, but the median expected *p*-value decreases 7200-fold (*p* = 1 × 10^–18^ to *p* = 1.5 × 10^–22^). Thus even for studies well-powered at 4 controls/case, increasing to 50 controls/case further reduces median expected *p*-values by 75-fold to 7000-fold, reducing below a “stringent” *p* = 3 × 10^–12^, providing concomitantly greater reassurance in the association.

Although increasing to 10–20 controls/case achieves most of the benefit of 50 controls/case, 50 controls/case typically represents a further reduction in the median expected *p*-value by factors of 2–10 versus 10–20 controls/case (Fig. 3; Table S2). If controls were truly low/no-cost, such reductions would generally be considered worthwhile. Little further reduction is achieved by 100 controls/case, unless α = 3 × 10^–12^.

### Fractional reduction, toward the null, in the minimum detectable odds-ratio with increasing number of controls/case

Using Eq. (3), Table 1 shows the reduction in the minimum detectable OR as the number of controls/case increases. Table 1 requires specifying only the minimum detectable OR at 1 control/case, which subsumes α, power, marker frequencies, and sample sizes. Table 1 can be referred to during study design to see the reduction in the minimum detectable OR by increasing the number of controls/case, regardless of α. Increasing from 1 to 4 controls/case reduces the minimum detectable OR toward the null by 20.9%, and from 4 to 50 controls/case reduces by an additional 9.7%. The maximum reduction is nearly achieved by 1000 controls/case, which achieves a 29.3% reduction versus 1 control/case and 10.5% reduction versus 4 controls/case.

**Table 1:** Minimum detectable OR from J controls/case, for example minimum detectable OR for 1 control/case, based on a derivation valid for small associations (see Supplement). Note that the percent reduction in the OR only depends on the number of controls per case, subsuming power, α, number of cases, or control marker frequency

Controls/case (J)	Minimum detectable odds-ratio (OR) for 1 control/case							% Reduction towards null vs J = 1 OR^a^	% Reduction towards null vs J = 4 OR^b^
	1.05	1.1	1.2	1.3	1.5	1.75	2		
1	1.05	1.1	1.2	1.3	1.5	1.75	2	0.0%	-26.5%
2	1.043	1.087	1.17	1.26	1.43	1.65	1.87	13.4%	-9.5%
3	1.041	1.082	1.16	1.25	1.41	1.61	1.82	18.4%	-3.3%
4	1.04	1.079	1.16	1.24	1.40	1.59	1.79	20.9%	0.0%
5	1.039	1.077	1.16	1.23	1.39	1.58	1.78	22.5%	2.0%
10	1.037	1.074	1.15	1.22	1.37	1.56	1.74	25.8%	6.2%
20	1.036	1.072	1.15	1.22	1.36	1.54	1.73	27.5%	8.3%
50	1.036	1.071	1.14	1.21	1.36	1.54	1.71	28.6%	9.7%
100	1.036	1.071	1.14	1.21	1.36	1.53	1.71	28.9%	10.1%
1000	1.035	1.071	1.14	1.21	1.35	1.53	1.71	29.3%	10.5%

^a^ Fractional reduction towards the null for minimum detectable odds-ratio for J controls/case (OR_J_) vs 1 control/case (OR_1_) equals (OR_1_ – OR_J_)/(OR_1_ – 1)

^b^ Fractional reduction towards the null for minimum detectable odds-ratio for J controls/case (OR_J_) vs 4 controls/case (OR_4_) equals (OR_4_ – OR_J_)/(OR_4_ – 1)

#### Example: considering the number of controls in GWAS

We have demonstrated the general value of recruiting > 4 controls/case for small α in Fig. 2, but in a specific situation, one must calculate the best number of controls/case to obtain. Here we consider genetic association studies. The Supplement details the allele-based power calculation and does not presume “local alternatives”. The calculation specifies the empirical OR between alleles and disease in a population, which is agnostic of genetic architecture except requiring Hardy–Weinberg Equilibrium [37].

To identify a minimum sample size where *p*-values have correct asymptotic performance even for rare minor allele frequencies (MAF), we simulated and calculated the skewness of test statistics and type-1 error (Supplement). We found that a minimum sample of 10,000 cases ensured validity of asymptotic *p*-values at rare marker prevalence of 1%, agreeing with prior work [38]. We will presume that enough cases have been chosen to ensure correct asymptotic performance of calculated *p*-values.

**Fig. 4:**
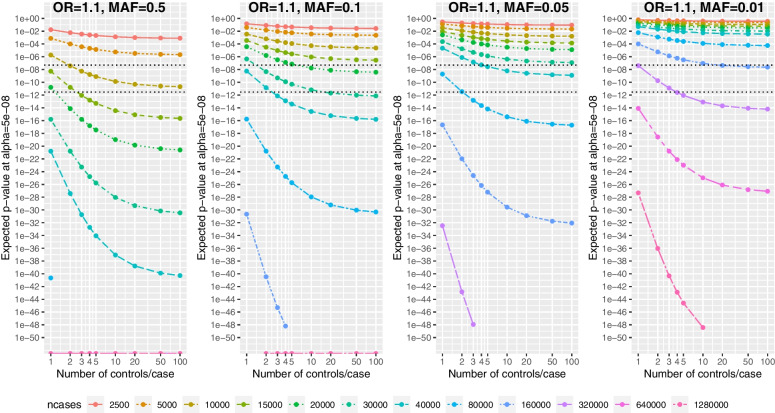
Expected *p*-value vs number of controls per cases, by the number of cases in a GWAS (α = 5 × 10^–8^) for OR = 1.1 and control minor allele frequency varying across plots from 0.5, 0.1, 0.05, 0.01. Dotted lines are expected *p* = α = 5 × 10^–8^ and “stringent” expected *p* = 3 × 10^–12^. *P*-values below 10^–50^ are not plotted. Both axes are on a log scale

Figure 4 shows the median expected *p*-value in GWAS (α = 5 × 10^–8^) by number of controls per case, at fixed number of cases at OR = 1.1 (Figure S1: OR = 1.2; Figure S2: OR = 1.05), and varying the control MAF from 0.5 to 0.01. The reduction in expected *p*-value with increasing controls/case is greatest when the *p*-values are smallest, and hence as the number of cases increases. Thus, the key result is that the reduction in expected *p*-value, by increasing controls/case, increases with more cases.

For example, increasing from 1 to 2 controls/case for OR = 1.1 and MAF = 0.5 reduces the expected *p*-value for 5,000 cases by 8-fold (*p* = 8 × 10^–4^ vs *p* = 1 × 10^–4^), for 10,000 cases by 50-fold (*p* = 2 × 10^–6^ vs *p* = 4 × 10^–8^), but for 30,000 cases by 100,000-fold (*p* = 2 × 10^–16^ vs *p* = 2 × 10^–21^). Similarly, increasing from 4 to 50 controls/case for OR = 1.1 and MAF = 0.5 reduces the expected *p*-value for 5,000 cases by 8-fold (*p* = 2 × 10^–5^ vs *p* = 3 × 10^–6^), for 10,000 cases by 60-fold (*p* = 2 × 10^–9^ vs *p* = 3 × 10^–11^), but for 30,000 cases by 200,000-fold (*p* = 2 × 10^–25^ vs *p* = 7 × 10^–31^). Note that extra controls provide greater reductions when expected *p* < α, which is helpful for attaining “stringent” *p-*values, such as *p* = 3 × 10^–12^, which provide greatest reassurance against reporting a false-positive association.

Table 2 shows how the minimum detectable OR at 80% power and α = 5 × 10^–8^ decreases with increasing numbers of controls/case, as control marker frequency varies from common to rare. Note that the fractional reductions, towards the null, by increasing number of controls/case are close to those of Table 1. Increasing beyond 10 controls/case does not seem to meaningfully reduce the minimum detectable OR.

**Table 2:** Minimum detectable OR at 80% power and α = 5 × 10^–8^ for a GWAS by control minor allele frequencies, number of controls/case, and number of cases

Number of cases	Number of controls/case		Minor allele frequency in controls			
		0.01	0.05	0.1	0.2	0.5
2,500 cases	1	2.74	1.68	1.48	1.35	1.29
	4	2.42	1.54	1.38	1.28	1.22
	10	2.35	1.51	1.35	1.26	1.21
	50	2.31	1.49	1.34	1.25	1.20
5,000 cases	1	2.12	1.46	1.33	1.24	1.20
	4	1.91	1.36	1.26	1.19	1.15
	10	1.86	1.34	1.24	1.18	1.14
	50	1.83	1.33	1.23	1.17	1.14
10,000 cases	1	1.74	1.31	1.22	1.17	1.134
	4	1.60	1.25	1.18	1.13	1.105
	10	1.57	1.23	1.17	1.12	1.098
	50	1.55	1.23	1.16	1.12	1.094
20,000 cases	1	1.50	1.216	1.155	1.116	1.093
	4	1.40	1.171	1.123	1.091	1.073
	10	1.38	1.161	1.115	1.086	1.068
	50	1.37	1.155	1.111	1.082	1.066
40,000 cases	1	1.34	1.150	1.108	1.081	1.065
	4	1.27	1.119	1.086	1.064	1.051
	10	1.26	1.112	1.080	1.060	1.048
	50	1.25	1.108	1.077	1.058	1.046
80,000 cases	1	1.24	1.105	1.076	1.057	1.046
	4	1.19	1.083	1.060	1.045	1.036
	10	1.18	1.078	1.056	1.042	1.034
	50	1.17	1.075	1.054	1.040	1.032
160,000 cases	1	1.165	1.074	1.053	1.04	1.032
	4	1.131	1.058	1.042	1.031	1.025
	10	1.123	1.055	1.04	1.03	1.024
	50	1.118	1.053	1.038	1.028	1.023
320,000 cases	1	1.115	1.052	1.037	1.028	1.022
	4	1.091	1.041	1.03	1.022	1.018
	10	1.086	1.038	1.028	1.021	1.017
	50	1.083	1.037	1.027	1.02	1.016

The Supplement derives two “rules of thumb” to assess the order of magnitude of the expected *p*-value attainable by increasing sample-size. First, the order of magnitude of the *p*-value at large controls/case equals roughly the square of the *p*-value at 1 control/case (“squaring rule”). Second, the median expected *p*-value at large controls/case is approximately equal to that of doubling the number of cases at 1 control/case (“doubling rule”). The “squaring” and “doubling” rules synergize to drive *p*-values below α. For example, consider the situation of where *p*-values of 10^–4^ have been observed at 1 control/case. Then the “doubling” rule implies that if the number of cases in the next GWAS were doubled, the *p*-value at 1 control/case would be approximately 10^–8^, and if large controls/case could be obtained, the “squaring” rule implies that the *p*-value could be approximately reduced to the order of 10^–16^. Thus increasing to a very large number of controls reduces the *p*-value in the same amount as doubling the number of cases (at 1 control/case).

### Example: reduction in *p*-values as the number of controls per case increases for 4 selected SNPs in the PLCO GWAS data

In Table 3, we chose individuals with SNPs genotyped on the Illumina Global Screening Array platform for melanoma (2093 cases), prostate cancer (2012 cases), and pancreatic cancer (578 cases) and 57,501 cancer-free controls in the PLCO GWAS [39] data. We then chose 50 random samples of controls at each controls/case ratio. Increasing from 4 to 10-25 controls/case reduced the *p*-value 108-fold and 991-fold (respectively; melanoma rs605965), 22-fold and 96-fold (respectively; melanoma rs871024), and fourfold and 13-fold (respectively; prostate cancer rs6983267). For pancreatic cancer rs635634, increasing from 4 to 10-25-50 controls/case reduced the *p*-value 14-fold, 36-fold, and 65-fold (respectively) and the fraction of the 50 random samples yielding genome-wide significance increased from 24% to 58%, 90%, and 100% (respectively). In these examples, increasing from 4 to 10 or 25 controls/case reduced the *p*-value up to 1–2 orders of magnitude and for 1 SNP (rs635634) increased the chance of achieving genome-wide significance from 24% to 58–90%.

**Table 3:** *P*-values for 4 SNPs as the number of controls per case increases in the PLCO GWAS Explorer data, by averaging 50 random samples of controls at each level of controls per case†. For the pancreatic SNP rs635634, we also calculate the percent of the 50 random samples that were statistically significant at *p* < 5 × 10^–8^

	Melanoma rs605965		Melanoma rs871024		Prostate rs6983267		Pancreas rs635634		
Controls per case	*P*-value	*P*-value ratio vs 4 controls per case	*P*-value	*P*-value ratio vs 4 controls per case	*P*-value	*P*-value ratio vs 4 controls per case	*P*-value	*P*-value ratio vs 4 controls per case	% Significant
1	2E-07		3E-07		2E-04		1E-04		2%
2	2E-09		3E-09		3E-05		5E-06		4%
3	7E-11		3E-10		7E-05		1E-06		14%
4	1E-11	1	6E-11	1	5E-05	1	4E-07	1	24%
5	1E-12	10	3E-11	2	4E-05	1	2E-07	2	30%
10	1E-13	108	3E-12	22	1E-05	4	3E-08	14	58%
25	1E-14	991	6E-13	96	4E-06	13	1E-08	36	90%
50	-	-	-	-	-	-	6E-09	65	100%
100	-	-	-	-	-	-	5E-09	85	100%

^†^ We chose individuals with SNPs genotyped on the Illumina Global Screening Array platform. We include the top 2 SNPs for melanoma (2093 cases; rs605965: OR = 1.47, MAF = 4.2%; rs871024: OR = 1.26, MAF = 49.6%), the top SNP for prostate cancer (2012 cases; rs6983267: OR = 0.86, MAF = 50%) and the top SNP for pancreatic cancer (578 cases; rs635634: OR = 1.48, MAF = 18.5%). There were 57,501 cancer-free controls and thus melanoma and prostate cancer had a maximum of 25 controls per case in the data

## Conclusions

We demonstrated that, as type-1 error α decreases, the increase in power as the controls/case ratio increases is larger, and the ratio reduction in the expected *p*-value is smaller, than for α = 0.05. Thus recruiting just 2 controls/case has increasingly greater value over 1 control/case as α decreases. At small α typical for thousands or millions of comparisons, versus 4 controls/case, recruiting 10–50 controls/cases can increase power, reduce the expected *p*-value by 1–2 orders of magnitude, and reduce the minimum detectable OR. Hence increasing controls/case could identify more novel associations and provide greater reassurance in previously reported associations. These benefits of increasing the controls/case ratio increase as the number of cases increases, although the amount of benefit depends on exposure frequencies and true OR. Although our example is genetic association studies, our findings apply to any study that uses small α to simultaneously assess many associations, such as -omic, consortial, or database studies.

We derived the fractional reduction, toward the null, of the minimum detectable OR for J controls/case versus 1 control/case, which asymptotes to a 29.3% reduction for large number of controls/case. This reduction depends only on specifying the minimum detectable OR for 1 control/case, regardless of the combination of α, power, marker frequencies, or sample sizes that imply that OR. Hence this reduction also applies to “regular” α = 0.05 epidemiology and, to our knowledge, has not previously been derived.

Our findings are consonant with a previous suggestion to use 10 controls/case in GWAS specifically for α = 1 × 10^–7^ [16]. However, the general point that power gains from raising the controls/case ratio increase with smaller α, to our knowledge, has not been previously recognized. We demonstrate that the percent reduction in expected *p*-value by raising controls/case increases with more cases. Thus the value of raising controls/case is greater when there are more cases, which, to our knowledge, has not been previously recognized. We provided rules of thumb to assess the order-of-magnitude of the expected *p*-value and the reduction in minimum detectable OR. Our findings apply to rare or common disease, are agnostic of genetic architecture (except requiring Hardy–Weinberg Equilibrium), and could be helpful to note in primers for GWAS study design [40].

Our calculations apply to the ideal scenario where controls are completely appropriate to the cases. If one borrows controls, small biases induced by borrowing inappropriate controls could negate power and *p*-value gains [41]. In exome/genome-wide analysis, at a minimum, there should be strict control for population structure [42]. Controls should be matched to cases with the same genotyping platform, the same variant calling, quality-control metrics and analysis pipeline, and imputed together with the same variants and reference panel [43]. Close attention should be paid to possible structural differences in the data caused by different laboratories and/or study populations [17–20]. These issues are generic to borrowing controls, irrespective of whether one borrows 1 or 100 control(s)/case.

A particular issue for GWAS is the importance of using the same genotyping platform for cases and controls to ensure comparable genotyping. If the GWAS has access to plentiful comparable controls, but which have been genotyped on a different platform, re-genotyping cases with the same platform as the plentiful controls could meaningfully increase power. This may be particularly useful for rare diseases, where it is hard to increase power by simply collecting many more cases.

Our calculations apply to planning a single study, not a discovery-replication 2-stage study that must account for “winner’s curse” and other issues [44]. Agreeing with prior work [45, 38, 22], we found that when there are few cases exposed to a rare marker, simple asymptotics may not apply and increasing the number of controls will not remedy the situation. When this situation occurs, more precise analysis is required [46].

We note that choice of α is crucial. Although Bonferroni adjustments are popular, they can be too conservative when test statistics are correlated. One approach is to use a parametric bootstrap on real data to assess the false-positive rate to determine α [12]. More research in this area is necessary.

Finally, determining the optimum number of controls is especially important for relatively understudied populations, for whom there are fewer cohorts/biobanks to borrow controls from. Our findings suggest that cohorts/biobanks of understudied populations should consider testing all controls to promote sharing of all appropriate controls across large-scale association studies for understudied populations.

## Supplementary Information

**Additional file 1.**

## Data Availability

Contact the Corresponding Author to obtain R programs.
