# Supplementary material for: Increase in power by obtaining 10 or more controls per case when type-1 error is small in large-scale association studies
Source: BMC Med Res Methodol. 2023 Jun 29;23:153. doi: 10.1186/s12874-023-01973-x (PMC10308790; doi:10.1186/s12874-023-01973-x)
Supplement: Supplementary file 1 — Additional file 1. [file 12874_2023_1973_MOESM1_ESM.docx]

**Online Supplement**

**Supplemental methods**

**Figure 1: Classical power calculations for small associations (“local alternatives”)**

The standard power/sample-size equation relating the detectable association (𝜇), two-sided size 𝛼, power 1-𝛽, and $\sigma_{0}$,$\sigma_{A}$ as the standard errors of the estimated 𝜇 under the null and alternative hypotheses, respectively, is equation 3.18 in (7):

$$\mu= Z_{\left\{ 1-\alpha/2 \right\}}\sigma_{0}+Z_{\left\{ 1-\beta\right\}}\sigma_{A},$$

where $Z_{\left\{ x \right\}}$ is the upper two-sided standard normal deviate at level $x$; for example $Z_{\left\{ 1-(5e-08)/2 \right\}}=5.45$ and $Z_{\left\{ 0.8 \right\}}=0.84$.

Under local alternatives, the minimum detectable effect approaches the null as the sample size increases. That is, we assume that the true association is small. Under local alternatives, we can assume equal standard errors under null and alternative ($\sigma=\sigma_{0}=\sigma_{A}$) to define power:

|  | $Z_{\left\{ 1-\beta\right\}}=\mu/\sigma-Z_{\left\{ 1-\alpha/2 \right\}}.$ | (1) |
| --- | --- | --- |

The 𝜇/𝜎 is also known as the “noncentrality parameter”. The local alternatives asymptotic is particularly relevant for studies that increase sample sizes to find ever-smaller associations, such as genome-wide association studies. Also, as we will see, local alternatives enables us to express the power of a study with J controls/case as a function of a study with 1 control/case, without needing to specify the numbers of cases or controls, variant prevalence, or effect-size.(1–7)

We consider the question, given that a study with 1 control/case has power 1-𝛽_1_, and standard error 𝜎_1_, what is the power 1-𝛽_J_ of a study with J controls/case (and standard error 𝜎_J_)? The power for each study is

$$Z_{\left\{ 1-\beta_{1} \right\}}=\mu/\sigma_{1}-Z_{\left\{ 1-\alpha/2 \right\}} and Z_{\left\{ 1-\beta_{J} \right\}}=\mu/\sigma_{J}-Z_{\left\{ 1-\alpha/2 \right\}}.$$

Under local alternatives, for a binary exposure, the approximate ratio increase in the variance of a matched design with J controls versus an infinite number of controls is $(J + 1)/J$.(2) Thus the ratio of variances from J controls/case relative to 1 control/case is

|  | $\frac{\sigma_{J}^{2}}{\sigma_{1}^{2}}=\frac{(J+1)/J}{2/1}=\frac{J+1}{2J}.$ | (2) |
| --- | --- | --- |

Solving this expression for $\sigma_{J}$, substituting into the power equation for J controls/case, and then combining with the power equation for 1 control/case, yields the classical power calculation for J controls/case given power for 1 control/case

|  | $Z_{\left\{ 1-\beta_{J} \right\}}=\left( Z_{\left\{ 1-\beta_{1} \right\}}+Z_{\left\{ 1-\alpha/2 \right\}} \right)\sqrt{\frac{2J}{J+1}}-Z_{\left\{ 1-\alpha/2 \right\}}.$ | (3) |
| --- | --- | --- |

Note that in the classical power calculation, the number of cases, exposure frequency, and the effect size do not explicitly appear. These factors are subsumed inside the power for a 1 control/case study under the situation of local alternatives.

The power gain (on the Z-statistic scale) for J controls/case versus 1 control/case is

|  | $Z_{\left\{ 1-\beta_{J} \right\}}-Z_{\left\{ 1-\beta_{1} \right\}}=\left( {Z_{\left\{ 1-\beta_{1} \right\}}+Z}_{\left\{ 1-\alpha/2 \right\}} \right)\left( \sqrt{\frac{2J}{J+1}}-1 \right),$ | (4) |
| --- | --- | --- |

The power gain from having many controls is greater as $Z_{\left\{ 1-\alpha/2 \right\}}$ increases (i.e., as 𝛼 decreases). Thus the power gain from having J controls/case is greater for smaller 𝛼 than for larger 𝛼. To be explicit, the actual power gain (i.e. on the scale of power, not Z-statistic for power) is

|  | $\left( 1-\beta_{J} \right)-\left( 1-\beta_{1} \right)=\Phi\left( Z_{\left\{ 1-\beta_{1} \right\}}+\left( {Z_{\left\{ 1-\beta_{1} \right\}}+Z}_{\left\{ 1-\frac{\alpha}{2} \right\}} \right)\left\{ \sqrt{\frac{2J}{J+1}}-1 \right\} \right)-\left( 1-\beta_{1} \right),$ |  |
| --- | --- | --- |

where $\Phi$ is the CDF of the standard normal distribution.

Note that as the number of controls/case becomes large, the power gain (on the Z-statistic scale) versus 1 control/case asymptotes:

$$Z_{\left\{ 1-\beta_{J} \right\}}-Z_{\left\{ 1-\beta_{1} \right\}}\approx\left( {Z_{\left\{ 1-\beta_{1} \right\}}+Z}_{\left\{ 1-\alpha/2 \right\}} \right)\left( \sqrt{2}-1 \right).$$

**Median expected p-value for an association under study replication**

The replication probability is the probability that repeating the same study would achieve a statistically significant result for the association.(33) Under our assumptions, the replication probability equals the power 1-𝛽 in equation (1):

$$Z_{\left\{ 1-\beta\right\}}=Z_{\left\{ 1-p/2 \right\}}-Z_{\left\{ 1-\alpha/2 \right\}},$$

where $Z_{\left\{ 1-p/2 \right\}}=\mu/\sigma$ is the noncentrality parameter, or “expected Z-statistic” (where 𝜇 is the true association with true standard error 𝜎), with corresponding 2-sided p-value denoted $p$. The expected Z-statistic is that expected under hypothetical study replications. The expected Z-statistic also equals the product of the noncentrality parameter and the square-root of sample size. The expected p-value corresponding to the expected Z-statistic solves for $p$:

$$p=2\times\Phi(-{|Z}_{\left\{ 1-\beta\right\}}+Z_{\left\{ 1-\alpha/2 \right\}}|),$$

where $\Phi$ is the CDF of the standard normal distribution.(33) We call this the “expected p-value”, but this is shorthand for “p-value corresponding to the expected Z-statistic over hypothetical replications”. That is, directly averaging p-values over hypothetical replications does not yield the above expression for $p$ because of the nonlinear inverse-probit function. Directly averaging small p-values would be heavily weighted toward the largest p-value. Instead, the proper scale is to average on the Z-statistic (or probit) scale, then transform that average to the p-value scale via the inverse-probit function.

This calculation of the expected p-value assumes that the true association 𝜇 and its true standard error 𝜎 equal their observed estimates and that normality applies.(33) Corrections exist for these issues, but the calculation is complicated.(35)

Note that the median expected p-value over hypothetical study replications is solely a function of 𝛼 and power.(33) For an association with 50% power, the expected p-value equals 𝛼. At 80% power and 𝛼=0.05, the expected p-value is 0.0051, and at 90% power, the expected p=0.0012. Vice versa, for an association with 10% power and 𝛼=0.05, the expected p=0.5. The expected p-value informs about the p-value that can be expected for an association with given power at 𝛼.

In addition to being an informative measure by itself, the expected p-value also demonstrates the value of seemingly small increases in power beyond 90%. For example, for 𝛼=0.05, increasing power from 90% to 99% reduces the expected p-value from 0.0012 to 0.000018, a 67-fold reduction in the p-value. For genome-wide significant 𝛼=5e-08, increasing power from 99% to 99.9% reduces the p-value by a factor of 570 (from p=7.4e-15 to p=1.3e-17).

The gain in the Z-statistic for the expected p-value $p_{J}$ from J controls/case versus the expected p-value $p_{1}$ from 1 control/case

$${Z_{\left\{ 1-p_{J}/2 \right\}}-Z}_{\left\{ 1-p_{1}/2 \right\}}=Z_{\left\{ 1-\beta_{J} \right\}}-Z_{\left\{ 1-\beta_{1} \right\}}$$

equals the power gain (on the Z-statistic scale) in equation (4). Note that small gains in power beyond power>0.9 will still yield a large power gain on the Z-statistic scale and hence a large ratio reduction in the expected p-value. For example, going from power of 0.96 to 0.99 is a difference on the Z-statistic scale of $Z_{\left\{ 0.99 \right\}}-Z_{\left\{ 0.96 \right\}}=2.33-1.75=0.58$, representing a substantial increase. Going from power of 0.99 to 0.999 is a difference on the Z-statistic scale of $Z_{\left\{ 0.999 \right\}}-Z_{\left\{ 0.99 \right\}}=3.09-2.33=0.76$, representing an even greater increase.

**Fractional reduction, toward the null, in the minimum detectable OR from 1 control/case to J controls/case, at any power and 𝛼, for ORs near the null (“local alternatives”)**

We consider the question, fixing 𝛼 and power at 1-𝛽, then what is the fractional reduction in the minimum detectable effect 𝜇_1_ from 1 control/case that can be gained by J controls/case 𝜇_J_? The power for each study is

$$Z_{\left\{ 1-\beta\right\}}=\mu_{1}/\sigma_{1}-Z_{\left\{ 1-\alpha/2 \right\}} and Z_{\left\{ 1-\beta\right\}}=\mu_{1}/\sigma_{J}-Z_{\left\{ 1-\alpha/2 \right\}}.$$

Thus

$$\frac{(Z_{\left\{ 1-\beta\right\}}+Z_{\left\{ 1-\alpha/2 \right\}})\sigma_{J}}{(Z_{\left\{ 1-\beta\right\}}+Z_{\left\{ 1-\alpha/2 \right\}})\sigma_{1}}=\frac{\mu_{J}}{\mu_{1}}$$

and plugging in (2) under local alternatives

$$\frac{\mu_{J}}{\mu_{1}}=\frac{\sigma_{J}}{\sigma_{1}}=\sqrt{\frac{J+1}{2J}} and hence \frac{\mu_{1}-\mu_{J}}{\mu_{1}}=1-\sqrt{\frac{J+1}{2J}}$$

Although 𝜇 is on the log(OR) scale, the 𝜇 is close to the null and hence $\mu\approx OR-1$. Thus the fractional reduction in the OR_1_, for ORs close to the null, is also

$$\frac{{OR}_{1}-{OR}_{J}}{{OR}_{1}-1}\approx1-\sqrt{\frac{J+1}{2J}}.$$

Note that as J controls/case increases, the fractional reduction in the minimum detectable OR at 1 control/case asymptotes at $1-\sqrt{1/2}=29.29\%$. Note that the fractional reduction in the minimum detectable OR is the same at any power, 𝛼, control marker frequency, and number of cases – these are all subsumed into the minimum detectable effect for J=1 control/case. The fractional reduction in the minimum detectable OR depends only on the number of controls/case.

Note that the fractional reduction toward the null, from doubling the number of cases and having also very large controls/case, equals $(1-\sqrt{1/2})(1+\sqrt{1/2})=50\%$.

**Standard power calculation for a genetic association study given specified control minor allele frequencies and odds-ratios**

Many power calculations have been proposed for GWAS, such as(13–15). The standard power calculation, valid for any alternative, is as follows. The variance under the alternative is

$$V_{1}=\frac{p_{1}(1-p_{1})}{2n}+\frac{p_{2}(1-p_{2})}{2nJ},$$

where $p_{1}$ and $p_{2}$ are the allele frequencies among cases and controls (respectively), $n$ is the number of cases, and $J$ is the number of controls/case. Note the factor of 2 is so that denominators are the number of alleles among cases and controls (note that an allele-based analysis requires Hardy-Weinberg Equilibrium to hold). It is convenient to write this variance as $V_{1}=\phi_{1}^{2}/(2N)$, where $N=2n(J+1)$ is the total number of alleles in cases and controls and

$$\phi_{1}^{2}=\frac{p_{1}(1-p_{1})}{1/(J+1)}+\frac{p_{2}(1-p_{2})}{J/(J+1)}.$$

Under the null hypothesis, the allele frequency does not differ between cases and controls and is equal to the marginal allele frequency

$$p_{0}=p_{1}\frac{1}{J+1}+p_{2}\frac{J}{J+1}.$$

The variance under the null is

$$V_{0}=p_{0}\left( 1-p_{0} \right)\left( \frac{1}{2n}+\frac{1}{2nJ} \right),$$

and it is convenient to write this as $V_{0}=\phi_{0}^{2}/(2N)$ where

$$\phi_{0}^{2}=p_{0}(1-p_{0})\left( \frac{1}{1/(J+1)}+\frac{1}{J/(J+1)} \right).$$

The standard power calculation is

|  | $Z_{\left\{ 1-\beta\right\}}=\frac{\sqrt{2N}\vert p_{1}-p_{2}\vert-{\phi_{0}Z}_{\left\{ 1-\alpha/2 \right\}}}{\phi_{1}}.$ | (5) |
| --- | --- | --- |

To calculate power, we specify control allele frequency p_2­_ and the OR and calculate case allele frequency p_1_ using

$$p_{1}=p_{2}+ \frac{\left( OR-1 \right)p_{2}(1-p_{2})}{1-p_{2}+OR\times p_{2}}.$$

Note that the specified OR is agnostic of the genetic disease model. That is, we specify the empirical OR observed in the population, without reference to a specific underlying genetic disease model.

To calculate the minimum detectable OR at power=0.8 for fixed 𝛼, we numerically solve power equation (5) for $p_{1}$ (specifying all other quantities) using the uniroot() function in R version 4.1.0. Then the minimum detectable OR is

$$OR=\frac{p_{1}/(1-p_{1})}{p_{2}/(1-p_{2})}.$$

We note that the [Genetic Association Study (GAS) Power Calculator](https://csg.sph.umich.edu/abecasis/gas_power_calculator/)(13,33) substitutes the variance under the alternative for the variance under the null (i.e substituting $\phi_{1}$ for $\phi_{0}$, see [GAS equation 15](http://csg.sph.umich.edu/abecasis/gas_power_calculator/equations_gas_power_calc.pdf)), which is appropriate for a Wald test. In our power calculations, we separately calculate the variance under the null and alternative hypotheses, which is akin to a score test or likelihood-ratio test. It is known that substituting the variance under the alternative is the reason that the Wald test can have low power to reject a null for a small sample and there is a large effect.(43,37) The score and likelihood-ratio are tests are preferable to the Wald test, especially at low minor allele frequencies.(22,37,43)

**Figure S3** plots the power for a Wald test (from GAS) versus the standard power calculation for a rare allele (MAF=1%) and 𝛼=5×10^-8^. Note that the greatest difference between the 2 power calculations is for large controls/case J, large RR for association, and few cases. This is because $\phi_{1}$ is dominated by the variance contribution from cases while $\phi_{0}$ is dominated by the variance contribution from controls. Thus substituting $\phi_{1}$ for $\phi_{0}$ underestimates power. The two power calculations converge as the number of cases grows, or as the RR goes towards 1.

**Evaluation of the validity of asymptotic p-values for rare markers at high control/case ratios**

When there are too few cases exposed to a rare marker, standard asymptotics may not apply and increasing the number of controls will not remedy the situation(43,37,22). Instead, the analysis must account for skewness of the test statistics.(44) We conducted 100M simulations for each control/case ratio (for 1k and 10k cases) with rare 1% control marker frequency at the null with 𝛼=5×10^-8^, for a score test. For 10k cases, the skewness asymptotes at only 0.069 at 100 controls/case, whereas for 1k cases the skewness asymptotes at a substantially higher 0.217 for 100 controls/case. For 10k cases, the type-I error is mildly inflated for J>4 controls/case (average: 1.2×10^-7^), but for 1k cases type-I error is substantially inflated (average: 9.3×10^-7^).

**“Squaring” and “Doubling” rules of thumb for considering the number of controls/case**

**Figures 3**, **S1**, and **S2**, and **Table 2** illustrate two handy “rules of thumb” (derived below). First, note that the order of magnitude of the p-value at very large controls/case equals roughly the square of the p-value at 1 control/case (“squaring rule”). Thus, because the p-value at 1 control/case decreases with the number of cases (fixing OR and marker frequencies), the expected p-value at very large controls/case is proportionately even smaller for as the number of cases increases.

Second, note that the median expected p-value at very large controls/case is approximately equal to that of doubling the number of cases at 1 control/case (“doubling rule”). Similarly, the minimum detectable OR with infinite controls/case equals that for double the number of cases but only 1 control/case: **Table 2** shows that the minimum detectable OR at n cases and 50 controls/case is very close to the OR for 2n cases and 1 control/case, for each control marker frequency. Hence doubling the number of cases is necessary to guarantee that the next study has lower expected p-values and lower minimum detectable OR.

The “squaring” and “doubling” rules help to approximate the sample size needed. For example, suppose that you have obtained all cases possible, and your power calculations show that the median expected p-value at 1 control/case were 10^-4^. By the “squaring” rule, you immediately know that at very large controls/case, the expected p-value would be on the order of 10^-8^, which is below genome-wide significance. In this situation, obtaining as many controls/case as possible would be crucial to attain genome-wide significance. Alternately, consider the situation of where many p-values of 10^-4^ have been observed at 1 control/case. Then the “doubling” rule shows that if the number of cases in the next GWAS were doubled, the p-value at 1 control/case would be 10^-8^, and if many controls/case could be obtained, the p-value would be reduced to the order of 10^-16^. The “squaring” and “doubling” rules synergize to drive p-values much below 𝛼.

Also, both doubling the number of cases and obtaining large numbers of controls/case, reduces the minimum detectable OR toward the null by 50%.

Derivation: “Squaring” rule of thumb (Figure 3): *As the number of controls/case becomes very large, the expected p-value is on the order of magnitude of the square of the expected p-value at 1 control/case.*

For small associations, the power for J controls/case, given that for 1 control/case is equation (3)

|  | $Z_{\left\{ 1-\beta_{J} \right\}}=\left( Z_{\left\{ 1-\beta_{1} \right\}}+Z_{\left\{ 1-\alpha/2 \right\}} \right)\sqrt{\frac{2J}{J+1}}-Z_{\left\{ 1-\alpha/2 \right\}}.$ |  |
| --- | --- | --- |

As J becomes very large, we have

|  | $Z_{\left\{ 1-\beta_{J} \right\}}+Z_{\left\{ 1-\alpha/2 \right\}}\approx\left( Z_{\left\{ 1-\beta_{1} \right\}}+Z_{\left\{ 1-\alpha/2 \right\}} \right)\sqrt{2}.$ |  |
| --- | --- | --- |

Defining the z-statistic for expected p-value with J controls/case as Z_J_, we have

$$Z_{J}\approx Z_{1}\sqrt{2}.$$

Thus as J becomes very large, the Z statistic for the expected p-value increases by a factor of the square-root of 2 over the Z statistic at 1 control/case. For large Z-statistics, the tail probability of a standard normal distribution is approximately

$$P(Z>z)\approx\frac{exp\{-z^{2}/2\}}{z\sqrt{2\pi}}.$$

Denoting the expected p-value at 1 control/case as p_1_, the expected p-value at very large number of controls/case p is

$$p=2P\left( Z_{J}>z_{1}\sqrt{2} \right)\propto p_{1}^{2}.$$

Thus the order of magnitude of the expected p-value at very large controls/case is roughly the square of the expected p-value at 1 control/case.

Derivation: “Doubling cases” rule of Thumb (Figure 3 and Table 2): *For OR near the null, when comparing a study with n cases and infinite controls/case to that of 2n cases and 1 control/case, (1) the expected p-value is the same, and (2) the minimum detectable OR is the same*

For OR near the null such that $p_{1}\approx p_{2}$, as J increases, the contribution to the variances from the controls disappears under the alternative

$$V_{1}=\frac{p_{1}(1-p_{1})}{2n}+\frac{p_{2}(1-p_{2})}{2nJ}\approx\frac{p_{1}(1-p_{1})}{2n}$$

and under the null

$$V_{0}=p_{0}\left( 1-p_{0} \right)\left( \frac{1}{2n}+\frac{1}{2nJ} \right)\approx\frac{p_{2}\left( 1-p_{2} \right)}{2n}.$$

If the number of cases is doubled, but J=1 control/case, then if $p_{1}\approx p_{2}\approx p_{0}$, we have

$$V_{1}=\frac{p_{1}(1-p_{1})}{2(2n)}+\frac{p_{2}(1-p_{2})}{2(2n)}\approx\frac{p_{1}(1-p_{1})}{2n}$$

and

$$V_{0}=p_{0}\left( 1-p_{0} \right)\left( \frac{1}{2(2n)}+\frac{1}{2(2n)} \right)\approx\frac{p_{2}\left( 1-p_{2} \right)}{2n}.$$

Thus the power calculation, and hence also the expected p-value and the minimum detectable OR, will be the same for n cases and infinite controls/case as for 2n cases and 1 control/case. Thus doubling the number of cases is required to be sure a study will have more power (or equivalently, smaller minimum detectable OR) than a study with half the number of cases but essentially unlimited controls. Note that this “rule of thumb” is true only for associations near the null.

**Supplemental Table S1.** Data plotted in Figure 1.

Power for a study with J controls/case when a study with 1 control/case study has power=0.1:

J 0.1 0.05 0.001 2.5e-06 5e-08 5e-09 3e-12

1 0.10 0.10 0.10 0.10 0.10 0.10 0.10

2 0.11 0.12 0.17 0.23 0.26 0.28 0.34

3 0.12 0.13 0.20 0.30 0.37 0.40 0.50

4 0.12 0.14 0.23 0.35 0.43 0.47 0.59

5 0.12 0.14 0.24 0.39 0.47 0.52 0.65

10 0.12 0.15 0.28 0.47 0.57 0.62 0.76

20 0.13 0.15 0.30 0.51 0.62 0.68 0.81

50 0.13 0.16 0.32 0.54 0.65 0.71 0.84

100 0.13 0.16 0.32 0.55 0.66 0.72 0.85

Power for a study with J controls/case when a study with 1 control/case study has power=0.2:

J 0.1 0.05 0.001 2.5e-06 5e-08 5e-09 3e-12

1 0.20 0.20 0.20 0.20 0.20 0.20 0.20

2 0.24 0.25 0.32 0.40 0.45 0.47 0.54

3 0.25 0.28 0.39 0.51 0.58 0.61 0.70

4 0.26 0.29 0.42 0.57 0.65 0.69 0.78

5 0.27 0.30 0.45 0.61 0.69 0.73 0.83

10 0.29 0.33 0.50 0.69 0.78 0.82 0.90

20 0.30 0.34 0.54 0.74 0.82 0.86 0.93

50 0.30 0.35 0.56 0.76 0.84 0.88 0.95

100 0.30 0.35 0.56 0.77 0.85 0.88 0.95

Power for a study with J controls/case when a study with 1 control/case study has power=0.3:

J 0.1 0.05 0.001 2.5e-06 5e-08 5e-09 3e-12

1 0.30 0.30 0.30 0.30 0.30 0.30 0.30

2 0.36 0.38 0.46 0.55 0.59 0.62 0.68

3 0.39 0.42 0.54 0.66 0.72 0.75 0.82

4 0.41 0.44 0.58 0.72 0.78 0.81 0.88

5 0.42 0.46 0.61 0.76 0.82 0.85 0.91

10 0.45 0.49 0.67 0.82 0.88 0.91 0.96

20 0.46 0.51 0.70 0.86 0.91 0.93 0.97

50 0.47 0.52 0.72 0.87 0.93 0.95 0.98

100 0.47 0.52 0.73 0.88 0.93 0.95 0.98

Power for a study with J controls/case when a study with 1 control/case study has power=0.5:

J 0.1 0.05 0.001 2.5e-06 5e-08 5e-09 3e-12

1 0.500 0.500 0.500 0.500 0.500 0.500 0.500

2 0.600 0.619 0.695 0.767 0.800 0.817 0.860

3 0.644 0.670 0.770 0.855 0.890 0.906 0.942

4 0.668 0.698 0.808 0.894 0.926 0.939 0.968

5 0.684 0.716 0.831 0.915 0.944 0.956 0.979

10 0.717 0.753 0.874 0.950 0.971 0.979 0.992

20 0.734 0.772 0.895 0.963 0.981 0.987 0.996

50 0.745 0.784 0.906 0.970 0.985 0.990 0.997

100 0.748 0.788 0.910 0.972 0.987 0.991 0.998

**Supplemental Table S2**. Data plotted in Figure 2.

Expected p-value for a study with J controls/case when a study with 1 control/case study has power=0.1:

J 0.1 0.05 0.001 2.5e-06 5e-08 5e-09 3e-12

1 0.7 0.5 0.040 6e-04 3e-05 5e-06 1e-08

2 0.7 0.4 0.020 8e-05 1e-06 1e-07 5e-11

3 0.7 0.4 0.010 3e-05 3e-07 2e-08 3e-12

4 0.6 0.4 0.010 1e-05 1e-07 8e-09 6e-13

5 0.6 0.4 0.009 1e-05 7e-08 4e-09 2e-13

10 0.6 0.4 0.007 4e-06 2e-08 7e-10 2e-14

20 0.6 0.3 0.006 2e-06 9e-09 3e-10 4e-15

50 0.6 0.3 0.005 2e-06 5e-09 2e-10 2e-15

100 0.6 0.3 0.005 1e-06 4e-09 1e-10 1e-15

Expected p-value for a study with J controls/case when a study with 1 control/case study has power=0.2:

J 0.1 0.05 0.001 2.5e-06 5e-08 5e-09 3e-12

1 0.4 0.3 1e-02 1e-04 4e-06 6e-07 8e-10

2 0.4 0.2 5e-03 8e-06 1e-07 7e-09 1e-12

3 0.3 0.2 3e-03 2e-06 2e-08 9e-10 6e-14

4 0.3 0.2 2e-03 1e-06 6e-09 2e-10 8e-15

5 0.3 0.1 2e-03 6e-07 3e-09 1e-10 2e-15

10 0.3 0.1 1e-03 2e-07 5e-10 1e-11 1e-16

20 0.3 0.1 7e-04 9e-08 2e-10 5e-12 2e-17

50 0.3 0.1 6e-04 6e-08 1e-10 2e-12 9e-18

100 0.3 0.1 6e-04 5e-08 9e-11 2e-12 6e-18

Expected p-value for a study with J controls/case when a study with 1 control/case study has power=0.3:

J 0.1 0.05 0.001 2.5e-06 5e-08 5e-09 3e-12

1 0.3 0.20 6e-03 3e-05 8e-07 1e-07 1e-10

2 0.2 0.10 1e-03 1e-06 1e-08 8e-10 9e-14

3 0.2 0.08 7e-04 3e-07 2e-09 7e-11 3e-15

4 0.2 0.07 5e-04 1e-07 5e-10 2e-11 3e-16

5 0.1 0.06 4e-04 7e-08 2e-10 6e-12 8e-17

10 0.1 0.05 2e-04 2e-08 3e-11 7e-13 3e-18

20 0.1 0.05 1e-04 8e-09 1e-11 2e-13 5e-19

50 0.1 0.04 1e-04 5e-09 5e-12 9e-14 2e-19

100 0.1 0.04 1e-04 4e-09 4e-12 7e-14 1e-19

Expected p-value for a study with J controls/case when a study with 1 control/case study has power=0.5:

J 0.1 0.05 0.001 2.5e-06 5e-08 5e-09 3e-12

1 0.10 0.050 1e-03 2e-06 5e-08 5e-09 3e-12

2 0.06 0.020 1e-04 5e-08 3e-10 1e-11 8e-16

3 0.04 0.020 6e-05 8e-09 2e-11 8e-13 1e-17

4 0.04 0.010 3e-05 3e-09 5e-12 1e-13 1e-18

5 0.03 0.010 2e-05 1e-09 2e-12 4e-14 2e-19

10 0.03 0.008 9e-06 2e-10 2e-13 3e-15 5e-21

20 0.02 0.007 6e-06 8e-11 5e-14 7e-16 6e-22

50 0.02 0.006 4e-06 4e-11 2e-14 3e-16 2e-22

100 0.02 0.006 4e-06 3e-11 2e-14 2e-16 9e-23

**Figure S1.** Expected p-value vs number of controls per cases, by the number of cases in a GWAS (𝛼=5×10^-8^) **for OR=1.2** and control minor allele frequency varying across plots from 0.5, 0.1, 0.05, 0.01. Dotted lines are expected p=𝛼=5×10^-8^ and “stringent” expected p=3×10^-12^. P-values below 10^-50^ are not plotted. Both axes are on a log scale.

**Figure S2.** Expected p-value vs number of controls per cases, by the number of cases in a GWAS (𝛼=5×10^-8^) **for OR=1.05** and control minor allele frequency varying across plots from 0.5, 0.1, 0.05, 0.01. Dotted lines are expected p=𝛼=5×10^-8^ and “stringent” expected p=3×10^-12^. P-values below 10^-50^ are not plotted. Both axes are on a log scale.

**Figure S3.** Number of controls/case versus power for a genetic association study (as calculated by GAS(13,45) or the standard 2-sample binomial calculation, detailed in the **Supplement**) where the minor allele frequency (MAF) is 0.01 (disease prevalence is 0.1), as RR decreases and number of cases increases, set so that at 1 control/case the power=0.1 at 𝛼=5×10^-8^. Black line is the power under local alternatives (i.e. as RR goes to 1 and number of cases goes to infinity). Y-axis is on a probit scale, x-axis is on a log scale.
